# Advanced treatment of secondary effluent from wastewater treatment plant by a newly isolated microalga *Desmodesmus* sp. SNN1

**DOI:** 10.3389/fmicb.2023.1111468

**Published:** 2023-01-26

**Authors:** Pengchong Wang, Yahui Shao, Yun Geng, Rubina Mushtaq, Wenlong Yang, Mei Li, Xiuqin Sun, Hongbo Wang, Gao Chen

**Affiliations:** ^1^Institute of Crop Germplasm Resources, Shandong Academy of Agricultural Sciences, Jinan, China; ^2^Shandong Provincial Key Laboratory of Crop Genetic Improvement, Ecology and Physiology, Jinan, China; ^3^School of Municipal and Environmental Engineering, Shandong Jianzhu University, Jinan, China; ^4^Institute of Molecular Biology and Biotechnology, The University of Lahore, Lahore, Pakistan

**Keywords:** secondary effluent, microalgae, biomass, nitrogen, phosphorus

## Abstract

Secondary effluents contain considerable amounts of nitrogen and phosphorous, which if dumped untreated can cause eutrophication of the receiving water bodies. Microalgae can remove these nutrients and other pollutants from the wastewater effluents and play an effective role in the secondary effluent treatment. In this study, six microalgae strains (SNN1, SNN2, SNN3, SNN4, SNS1, and SNS2) were isolated and screened from the water and mud of Yingxue Lake of Shandong Jianzhu University, and their efficiencies for the removal of COD, NH_4_^+^-N, TN, and TP in the secondary effluent were assessed. By comparing the growth performances and nutrient removal ability of algal strains in domestic sewage, we found that SNN1 (identified and named as *Desmodesmus* sp. SNN1) has the highest efficiency for biomass accumulation and sewage purification. Hence, the algal strain SNN1 was selected for further screening and optimization experiments. The strain showed higher biomass yield and better nutrient removal rate when the pH of secondary effluent was 9.0 and the initial inoculum concentration (optical density at 680 nm) of algal strain was 0.4. After 12 days of treatment, the concentrations of COD, NH_4_^+^-N, TN, and TP in the secondary effluent were 31.79, 0.008, 8.631, and 0.069 mg/L, respectively. Therefore, SNN1 with the removal rates of 52.69% (COD), 99.99% (NH_4_^+^-N), 89.09% (TN), and 94.64% (TP) displayed its high potential in nutrient removal. In addition, it also yielded 5.30 mg/L of chlorophyll a and 168.33 mg/L of lipids. These results demonstrated that this strain exhibited an effective treatment capacity for secondary effluent and microalgal oil production. This study is helpful to provide a strategy for the resource utilization of secondary effluent and the conservation of freshwater resources required by microalgae culture.

## 1. Introduction

Sewage treatment plants play an important role in improving water quality and efficient use of water resources. After sedimentation treatment, the effluent (primary effluent) is further treated to remove dissolved pollutants using biological resources, and is called secondary effluent (Hu et al., 2016). The secondary effluent produced by anaerobic-anoxic-aerobic (A^2^O) process, the most used sewage treatment process in China, is the effluent with lower organic matter, nitrogen, and phosphorus content. However, the management of surplus sludge generated in this process has always been a big challenge, as it escalates the operational and maintenance expenditure of the water treatment plants (Jin et al., 2014). Inorganic nitrogen, phosphorus, and other chemical compounds in secondary effluent produced after domestic sewage treatment cannot be effectively reduced and generate gaseous effluents such as CO_2_ and NO_x_. Production of such toxic substances pollutes the environment and poses health risks (Katam and Bhattacharyya, 2019). To avoid this, many countries have set their own standards for the discharge of wastewater in water bodies. For example, according to the Chinese National First A-level Sewage Discharge Standard, the secondary effluent to be discharged should not exceed the threshold of 50 mg/L chemical oxygen demand (COD), 5 mg/L ammonium (NH_4_^+^), 15 mg/L total nitrogen (TN), and 0.5 mg/L total phosphorous (TP; Lv et al., 2017).

Microalgae are single-celled autotrophic microorganisms that inhabit diverse environments for their higher photosynthetic efficiency, strong adaptability, and resilience to abiotic stress. Microalgae require carbon (C) in higher amounts, followed by nitrogen (N) and phosphorus (P) along with other macro-micronutrients for their growth (Cai et al., 2013). All these nutrients are richly available in secondary effluents. Various studies have reported that microalgae are effective in sequestering these nutrients, minerals, and heavy metals, and in reducing some organic compounds (Leite et al., 2019). Therefore, different microalgae strains are being used in the treatment of wastewater in a process called phycoremediation. Zhang et al. (2015) cultivated *Scenedesmus dimorphus* (microalga) in secondary effluent and reported its high potential in the removal of TN and TP. In addition, they also suggested that cultivation of microalgae in domestic secondary effluent (DSE) could be a cost-effective option in microalgae-based biodiesel production. This finding endorses other studies suggesting that the ability of microalgae to yield biodiesel makes them a good choice to deal with the reduction in fossil fuel reserves (Chisti, 2008; Gouveia and Oliveira, 2009). In a recent research, Lee et al. (2022) isolated *Chlorella sorokiniana* JD1-1, a freshwater microalga, and performed its growth studies in domestic sewage (DWW) and livestock wastewater (LWW). They determined that the strain showed a higher growth rate in DWW, with the removal rates of TN and TP up to 97 and 99%, respectively. Moreover, the recovered microalgae can be further utilized as biofertilizers and animal feeds (Zhu et al., 2013; Raheem et al., 2015). Indeed, phycoremediation has emerged as a sustainable, eco-friendly, cost-effective, and efficient alternative to conventional methods of wastewater reclamation (Posadas et al., 2015; Shahid et al., 2020).

Microalgae from culture collections have been evaluated for wastewater reclamation and biofuel production potential. However, quite a few attempts have been made to isolate and screen indigenous microalgal species (Abdelaziz et al., 2014). While indigenous microalgae strains have been detected to possess higher capacity of secondary effluent treatment with greater biomass production due to their high adaptability to the local environment (Lynch et al., 2015). Therefore, the isolation and characterization of strains from indigenous algal habitats can be a favorable exercise to obtain algal strains efficient in improving secondary effluent quality and producing compounds of industrial importance (Chen et al., 2015).

Microalgae *Desmodesmus* sp. is widely used in sewage treatment. Wang et al. (2022) concluded that the maximum removal rate of total nitrogen, total phosphorus, and chemical oxygen demand by *Desmodesmus* sp. in actual wastewater treatment exceeded 95%. Luo et al. (2018) showed that *Desmodesmus* sp. CHX1 performed well in nutrient removal from piggery wastewater, with ammonium nitrogen (NH_4_–N), and TP removal efficiency (RE) of 78.46 and 91.66%, respectively, after 7 days of culture. Furthermore, culturing microalgae *Desmodesmus* sp. to remove the contaminants in the wastewater was also helpful to produce biomass and lipid (Do et al., 2019; Hernández-García et al., 2019).

The algal strains used in our research were isolated from the water and mud of Yingxue Lake of Shandong Jianzhu University. The algae strains with strong adaptability in the secondary effluent were initially screened and purified. Further characterization of algal strains based on their potential to accumulate biomass and remove nutrients from the secondary effluent was conducted. The growth parameters for the selected microalgal strains were optimized to obtain higher biomass yield and better nutrient removal rates from the secondary effluent. This study provides the strategy to obtain higher biomass of microalgae with the purification of secondary effluent, simultaneously.

## 2. Materials and methods

### 2.1. Sample collection

The microalgae used in this work were isolated from the water and mud of Yingxue Lake in Shandong Jianzhu University. The secondary effluent from the reclaimed water station of Shandong Jianzhu University was used to culture microalgae. The secondary effluent quality indicators were as follows: chemical oxygen demand (COD) 60–90 mg/L, ammonia nitrogen (NH_4_^+^-N) 40–60 mg/L, total nitrogen (TN) 60–80 mg/L, total phosphorus (TP) 2–4 mg/L, and pH 7.4–7.6.

### 2.2. Culture conditions

For the isolation and maintenance of algal strains, BG-11medium was used as described elsewhere (Chen et al., 2021). To quantify efficiency of strains in biomass production and reclamation of water, secondary effluent from the above-described location was utilized. Generally, the strains were cultured in conical flasks (100 ml) filled with 50 ml of BG-11 medium or secondary effluent according to the requirement of the experiment. The cultures were incubated in an incubator fitted with fluorescent light (40 μmol photons·m^−2^·s^−1^) while the temperature range was 25–30°C (Chen et al., 2014). The flasks were manually shaken periodically throughout the incubation period.

### 2.3. Experimental methods

#### 2.3.1. Isolation and screening of algae strains

The lake mud was preliminarily diluted with sterilized water and then filtered with gauze to remove impurities and planktons. The lake water was directly filtered with gauze without dilution. The filtered lake water and lake mud samples were diluted with sterile water in a gradient of 10^−1^, 10^−2^, 10^−3^, and 10^−4^. 0.5 ml of diluted sample from each concentration was spread on BG-11 agar plates and incubated at 25–30°C with light of 40 μmol photons·m^−2^s^−1^. Algal colonies of different shapes and colors were selected and cultured again on the solid BG-11 agar plates for further purification. The process was repeated until single algal species were obtained. For further experiments, each strain was grown in 50 ml BG-11 medium for 8 days under the culture conditions as described above.

#### 2.3.2. Identification of algae strains

The algae strains were observed under a microscope, preliminarily judged, and distinguished according to its shape, color, and size. Further, the genomic DNA of each microalgal strain was extracted by the modified Cetyltrimethylammonium bromide (CTAB) method (Zhang et al., 2021). PCR amplification was performed with 18S rDNA primer sets 5′-CCTGGTTGATCCTGCCAGTAG-3′, 5′-TTGATCCTTCTGCAGGTTCA-3′ and 5′-TCCGTAGGTGAA CCTGCGG-3′, and 5′-TCCTCCGCTTATTGATATGC-3′. The PCR products were purified on 1% agarose gel and sequenced by commercial sequence providers (Qingdao Qingke Biotechnology Co., Ltd.) The sequences were further verified by TA cloning and analyzing complete sequences obtained using M13 sequence primers. Algal strains were identified through comprehensive analysis of the colony characteristics, cell morphology, and 18S rDNA sequencing results of the isolated and purified algae strains. The 18S rDNA sequences were searched for sequence similarity over NCBI nucleotide BLAST server,^1^ and phylogenetic tree was constructed by neighbor-joining method in MEGA 11 software (Tamura et al., 2021).

#### 2.3.3. Selection of algae strains

The selected algae were cultured in the secondary effluent under the conditions as described above (section 2.2) for 12 days. Samples were taken every 2 days and COD, NH_4_^+^-N, TN, TP, and other water quality indicators of the secondary effluent were measured. To estimate the biomass, the optical density at 680 nm (OD_680_) of each algal strain was measured using UV–visible spectrophotometer (TU-1800, Beijing Purkinje). Biomass accumulation and water quality parameters (COD, NH_4_^+^-N, TN, and TP) of the secondary effluent treated with different algal strains were quantified using methods as described elsewhere (Ji et al., 2021).

#### 2.3.4. Influence of initial pH of secondary effluent

Five groups of secondary effluents with the initial pH of 5.0, 6.0, 7.0, 8.0, and 9.0 were set for the experiment. Two replicates were used for each group of experiments. The initial cell density of 0.2 (OD_680_ value) was cultured and incubated under light intensity of 40 μmol photons·m^−2^s^−1^ at 25–30°C for 12 days. Water quality index and biomass index of the experimental groups were quantified every 2 days of incubation.

#### 2.3.5. The effect of different initial OD values on secondary effluent reclamation by microalgae

Microalgae were inoculated into the secondary effluent in different initial cell densities (OD_680_ values). According to the growth of microalgae and the treatment of sewage, the most appropriate initial OD value of microalgae was selected. The initial OD value gradient was 0.1, 0.2, 0.4, and 0.6. Each concentration was analyzed in two parallel experiments. The experimental light intensity was 40 μmol photons·m^−2^s^−1^, temperature of incubation room was 25–30°C with 24 h light incubation for 12 days. Initial pH of secondary effluent used was the optimum pH value obtained by the analysis of effect of initial pH of the secondary effluent. Samples were collected every 2 days to determine the biomass growth, chlorophyll a (Chl a) content, lipid yield and the concentrations of TN, TP, NH_4_^+^-N and COD.

### 2.4. Analysis method

#### 2.4.1. Determination of water quality indicators

Each sample was collected in a 50 ml falcon tube and centrifuged at 7,000 rpm for 10 min. The supernatant was separated and filtered through a 0.45 μm pore diameter filter membrane to remove algal cells. The filtrate was used to quantify the sewage quality indicators, such as COD, TN, and TP. All analyses were conducted according to the Standard Method for the Examination of Water and Wastewater (Baird et al., 2012), while the following formula was used to calculate the removal rates of TN, NH_4_^+^-N, TP, and COD:


(1) $$\mathrm{RR}=\left(C_{0}-\mathrm{Ct}\right)/C_{0}$$

where RR: removal rate (%); C_0_: initial concentration (mg/L); and Ct: time concentration (mg/L).

#### 2.4.2. Biomass

To determine the microalgae biomass in the sewage group, the light density method was used as described elsewhere (Santos-Ballardo et al., 2015). Briefly, cultures were incubated in secondary effluent under the similar culture conditions as described above for 12 days. Samples were taken every 2 days, and OD_680_ was measured using the same UV–vis spectrophotometer for each sample. The recorded cell density values were used to draw growth curves.

#### 2.4.3. Chlorophyll a

To determine chlorophyll a content, 5 ml of algal culture was taken in a 10 ml centrifuge tube and centrifuged at 6,000 rpm for 15 min, and the supernatant was discarded. The pellet was resuspended in 5 ml of 95% ethanol solution, mixed evenly, and left at 4°C for 24 h in the dark for maximum pigment extraction. The suspension was centrifuged at 8,000 rpm for 10 min, the supernatant was collected, and its optical density was measured at 649 and 665 nm with a UV–vis spectrophotometer. Chl a content was measured according to the following formula (Zhang et al., 2019).


(2) $$\mathrm{Chlorophyll}\;a=13.95\times A_{665}-3.88\times A_{649}$$

#### 2.4.4. Microalgae lipid extraction

The improved chloroform methanol method was used to determine the lipid content in microalgae, as described elsewhere (Abomohra et al., 2016). For lipid extraction, 30 ml of algal culture was taken in a 50 ml falcon tube. The tube was centrifuged at 7,000 rpm for 10 min and the supernatant was discarded. The pellet was resuspended in 500 μl of 1 mol/L hydrochloric acid solution. The tube was vortexed on the vortex oscillator to break the cell walls of microalgae, and chloroform/methanol (volume ratio 2:1) solution was added to it. For maximum extraction, the tube was placed in a dark shaking incubator (150 rpm) for 4 h. After incubation, 10 ml normal saline solution was added to the test tube, vortexed for 60 s, and centrifugated at 7,000 rpm for 10 min. After centrifugation, chloroform phase was shifted to the aluminum foil tin plates with the help of a syringe. Then, the tin plates were put at 60°C in an oven to get the constant mass.


(3) $$C=\frac{M_{1}-M_{0}}{V}$$

(4) $$\eta =\frac{C}{M}$$

where *η*: Lipid content, (%); *C*: Lipid yield, (g/L); *M_0_* and *M_1_* are the mass of the aluminum foil tin plate without and with lipid extracts after drying, respectively, (g); *V*: measured volume of algal liquid, (L); and *M*: dry weight of microalgae, (g/L).

#### 2.4.5. Statistical analysis

Statistical analysis was performed by one-way ANOVA. *p* values of 0.05 or less were considered statistically significant. Error bars represent the SD of the biological replicates from a single experiment. The data were analyzed using Origin 2021 and GraphPad prism 9.

## 3. Results and discussion

### 3.1. Isolation and identification of algae strains

**Figure 1:**
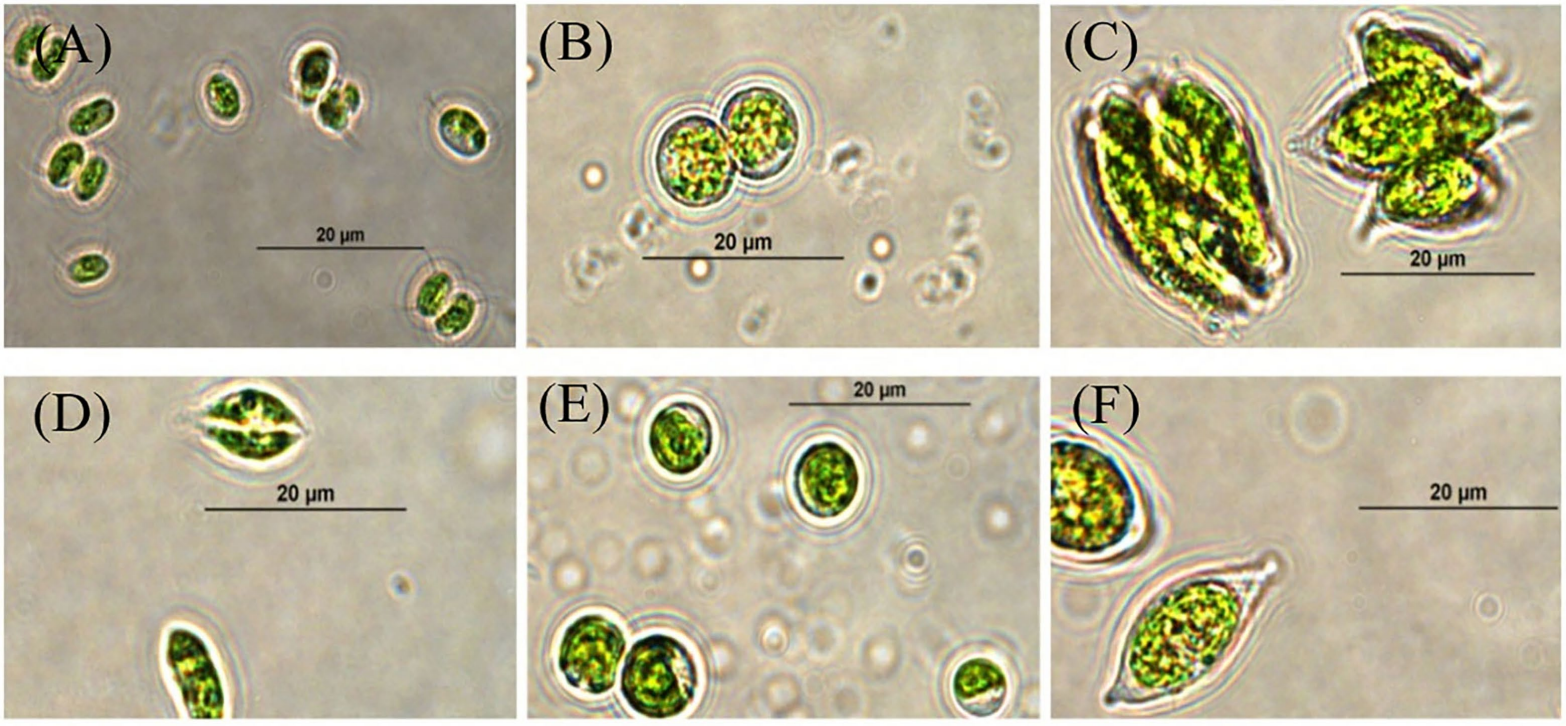
Micrographs of selected strains of algae visualized and photographed under a compound microscope (100X). **(A)** SNN1; **(B)** SNN2; **(C)** SNN3; **(D)** SNN4; **(E)** SNS1; and **(F)** SNS2.

Six microalgae strains were isolated and purified. Among them, four algae strains (SNN1, SNN2, SNN3, and SNN4) were isolated from lake mud. Two algae strains (SNS1 and SNS2) were isolated from the lake water. The analysis of micrographs of the strains was performed using the morphological characteristics of microalgae strains in literature (Rani et al., 2022). The strains were found to have close resemblance with *Chlorella* sp. and *Scendesmus* sp. as shown in Figure 1.

However, the analysis of the sequence results of BLAST showed that the strains were four different species of algae. Out of these algal strains, SNN3, SNN4, and SNS2 showed maximum similarity to a single species of algae. Hence, SNN4 was arbitrarily chosen for further experiments. Evolutionary relationship analysis of the four species of algae through phylogenetic tree depicted that SNN1 was in common clade with *Desmodesmus* sp. XFLZ928.6; strain SNN2 shared a clade with *Coelastrella rubescens* var. *oocystiformis* isolate FACHB-2297; strain SNN4 was in clade with *Scotiellopsis reticulata* strain CCALA 474; and strain SNS1 shared clade with *Chlorella sorokiniana* strain ACSSI 364 (Supplementary Figure S1). Based on morphological characteristics and 18S rDNA sequence similarity results, strain SNN1 was named as *Desmodesmus* sp. SNN1.

### 3.2. Screening of target algae strains

#### 3.2.1. Analysis of secondary effluent treatment by different microalgae

**Figure 2:**
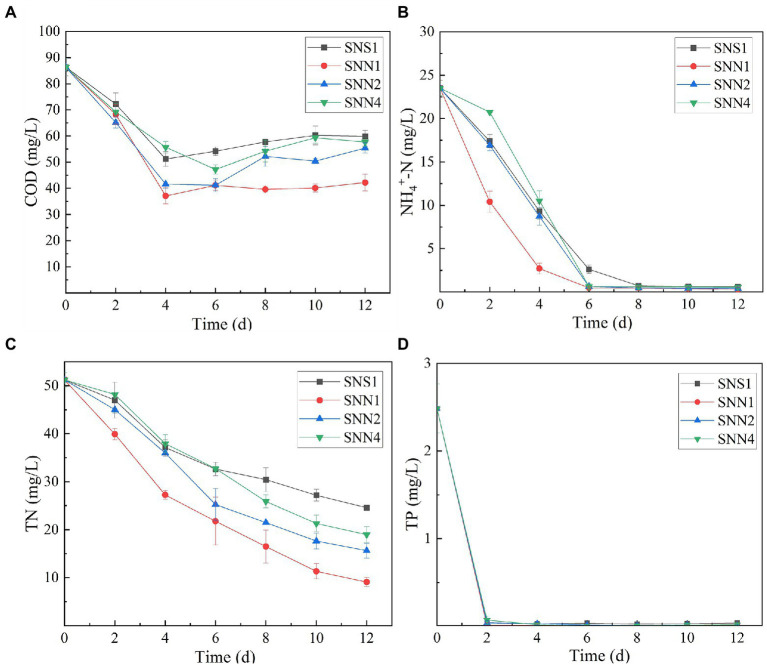
Nutrient removal from secondary effluent by algal strains during the culture incubation time. **(A)** COD; **(B)** NH_4_^+^-N; **(C)** TN; and **(D)** TP.

Wastewater treatment is a burden on economies. However，if it is used for the cultivation of microalgae and cyanobacteria, it may not only save the pretreatment costs rather it will be considered as a low-cost resource (Shahid et al., 2021a). Algal strains exhibited different indices for the different pollutants removed from the secondary effluent (Figure 2). The removal rate of COD and TN was low, while the removal rate of phosphorus and NH_4_^+^-N was relatively higher. After 12 days of treatment, SNN1 showed the highest COD removal rate of 51.1%. In addition, till the eighth day of culture incubation, all strains almost completely consumed NH_4_^+^-N present in the secondary effluent. The removal rate of NH_4_^+^-N by SNN1 was the highest, while SNN4 exhibited the lowest NH_4_^+^-N removal rate (Figures 2A,B; Table 1). Microalgae can assimilate ammonia in wastewater and convert this ammonia into amino acid glutamine (Liu et al., 2017). The complete removal of NH_4_^+^-N from treated secondary effluent by four microalgae strains showed that these isolated algae species, especially SNN1, had higher potential to assimilate ammonium into cells as described elsewhere (Sinclair et al., 2009).

**Table 1:** Treatment effect and biomass accumulation of different algae strains in secondary effluent.

Algae strains	Removal rate (%)				Biomass
	COD	NH_4_^+^- N	TN	TP	OD_680_
SNS1	30.68 ± 1.33	97.43 ± 0.32	52.05 ± 0.27	98.51 ± 0.16	1.224 ± 0.084
SNN1	51.13 ± 1.85	98.62 ± 0.53	82.26 ± 0.96	99.15 ± 0.20	1.803 ± 0.036
SNN2	35.88 ± 1.09	98.23 ± 0.28	69.49 ± 1.51	99.44 ± 0.14	1.208 ± 0.088
SNN4	33.23 ± 0.93	97.63 ± 0.40	62.97 ± 1.61	99.24 ± 0.16	1.373 ± 0.061

Data are shown as their replicate mean ± SD.

All four test strains were recorded to have different TN removal efficiency from secondary effluent. The TN removal rate was the highest (82.26%) for strain SNN1, followed by the almost similar removal rates of 69.49 and 62.97% displayed by SNN2 and SNN4, respectively. Least TN absorption ability (52.05%) was recorded for SNS1 (Figure 2C). However, all the strains removed phosphorous efficiently, and complete removal was detected by the fourth day of culture incubation (Figure 2D). Our finding is strengthened by the previous report that *Scenedesmus* sp. exhausted almost all phosphorus when cultured in modified BG 11 medium containing nitrogen and phosphorus sources (Li et al., 2010).

#### 3.2.2. Analysis of growth of different microalgae in secondary effluent

**Figure 3:**
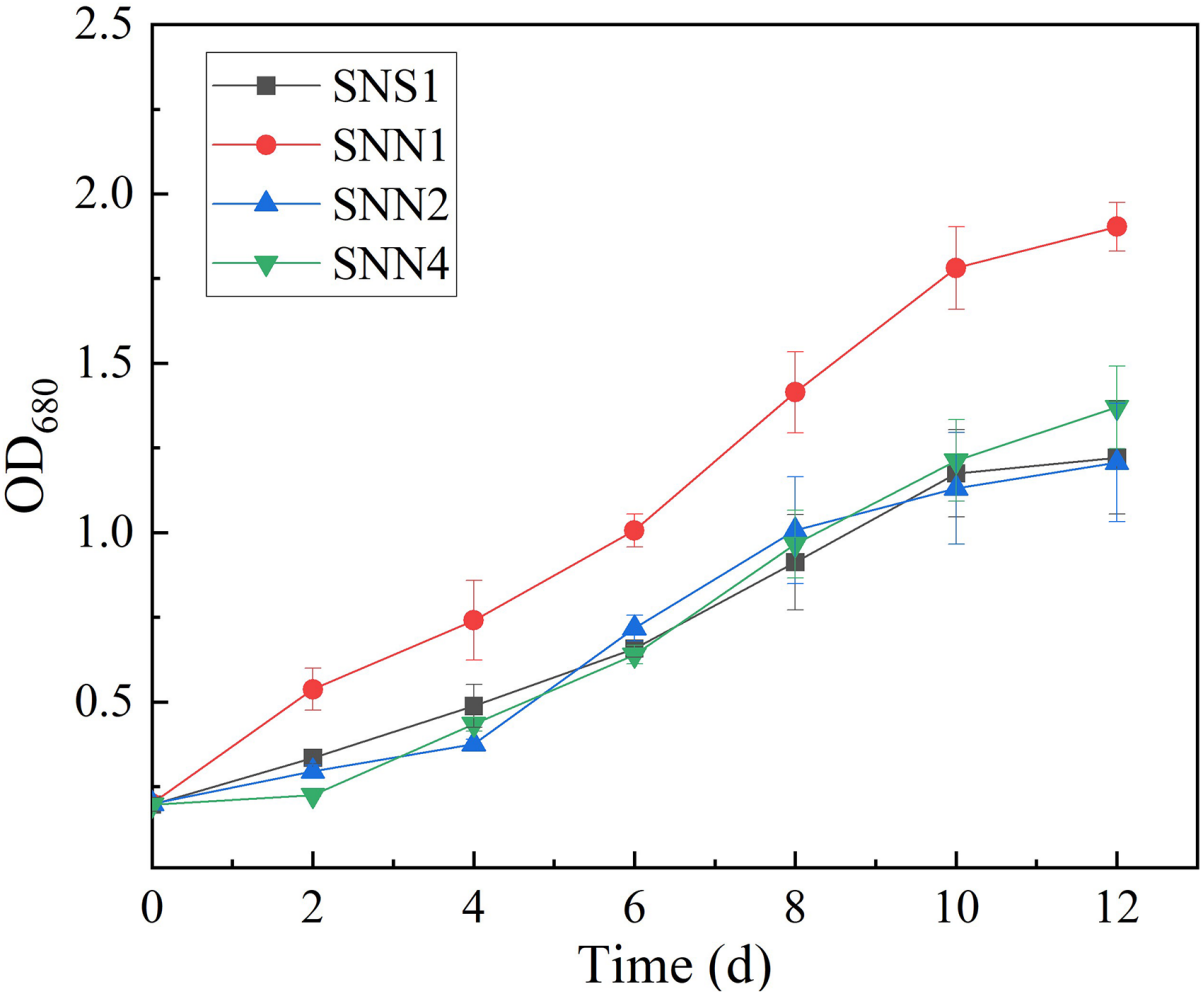
Cell density (OD_680_) of four microalgae strains in secondary effluent during the 12 days of culture incubation measured by UV–vis spectrophotometer.

The quantification of biomass growth of the four strains in the secondary effluent showed that SNN1 grew well in the sewage and displayed strong anti-pollution performance. It attained OD_680_ of 1.803, which was maximum among all the strains characterized. Although SNS1, SNN2, and SNN4 also maintained exponential growth, their biomass yield was much lower than the SNN1 (Figure 3). In the process of using wastewater for microalgae culture, it is important to select and optimize the appropriate strains. Previous studies have extensively studied the screening and separation of new microalgae species to achieve efficient aquaculture and better nutrient removal efficiency (Shen et al., 2020; Ye et al., 2020). Chen et al. (2020) isolated *Desmodesmus* sp. from local pig farm wastewater in Fujian Province, China, and reported its ability to treat pig farm wastewater. In addition, Ling et al. (2019) reported that the productivity of microalgae biomass and wastewater removal efficiency can be significantly improved by using locally isolated microalgae strains. Our experimental results showed that the isolated algal strain SNN1 achieved better biomass accumulation during the cultivation in wastewater and was more suitable for growth and cultivation in secondary effluent.

The analysis and comparison of the treatment effects and biomass accumulation of algal strains showed that the strain SNN1 had the higher nutrient removal rate and biomass accumulation than those of SNS1, SNN2, and SNN4 (Table 1). These findings suggest that SNN1 possessed strong adaptability to the environment, good growth capacity, and greater potential for wastewater treatment. Therefore, we selected this strain for our further experiments.

### 3.3. Impact of environmental conditions on secondary effluent treatment by microalga

#### 3.3.1. Initial pH

**Figure 4:**
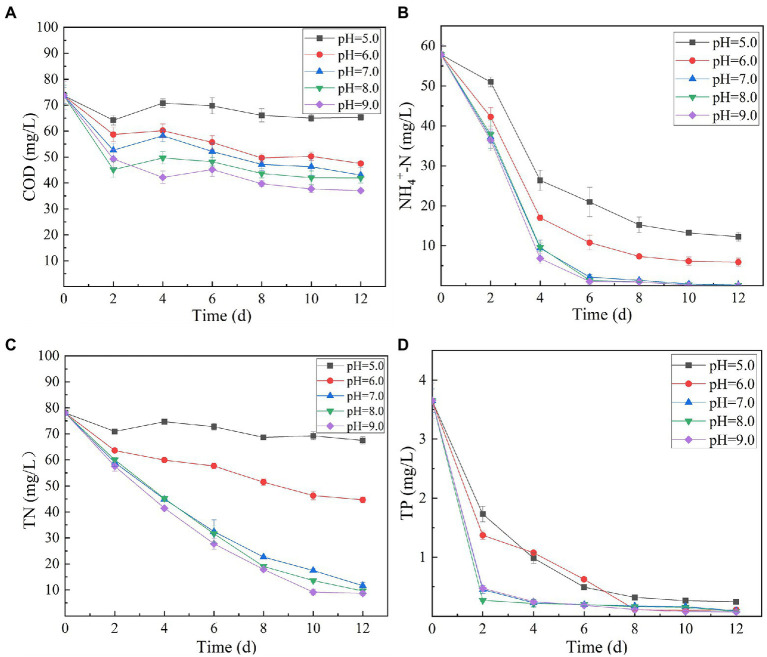
Effect of different initial pH (5.0–9.0) of secondary effluent on the nutrient removal efficiency of algal strain SNN1 during 12 days of culture incubation. **(A)** COD; **(B)** NH_4_^+^-N; **(C)** TN; and **(D)** TP.

Variation in initial pH of secondary effluent had a significant effect on the efficiency of algal strain SNN1 in wastewater treatment (Table 2; Figure 4). The highest capacity of SNN1 for secondary effluent treatment was recorded at pH 9.0 with COD, NH_4_^+^-N, TN, and TP removal/degradation rates of 49.80, 99.95, 88.90, and 97.92%, respectively (Table 2). At the initial pH 5.0 of the secondary effluent, the removal rates of COD, NH_4_^+^- N, TN, and TP by this algal strain were significantly reduced. These results showed that the high alkaline environment (pH 9.0) was more conducive to the removal of nutrients. The inhibition of removal of nutrients under acidic environment might be attributed to the fact that low pH affects the nutrient absorption strength of microalgae and also reduces the activities of enzymes related to photosynthesis (Sari et al., 2013). Experiments show that alkaline conditions are more suitable for the survival of algae strains. The optimum pH values for the growth of different algae strains are different. When deviating from the optimum pH value, the growth of the algal strain and the metabolic activities in the body will be inhibited. pH 9.0 and pH 8.0 are both alkaline, but pH 9.0 is more suitable for the survival of microalgae. Our results are consistent with the findings of Ma et al. (2016), who reported that alkaline conditions enhanced nutrient removal capacity of microalgae. Therefore, SNN1 can be effectively employed in the reclamation of weakly alkaline wastewater, for example, for the treatment of wastewater generated during the synthesis and printing of paper (Jagaba et al., 2022; Liu et al., 2023).

**Table 2:** Effects of initial pH and initial inoculation amount on biomass and nutrient removal efficiency of algal strain SNN1.

Factor	Level	COD removal efficiency (%)	NH_4_^+^-N removal efficiency (%)	TN removal efficiency (%)	TP removal efficiency (%)	OD_680_ accumulation	Accumulation of chlorophyll a (mg/L)
Initial pH	5.0	11.39 ± 0.81	78.79 ± 0.96	13.62 ± 0.88	93.23 ± 0.45	0.431 ± 0.011	1.36 ± 0.10
	6.0	35.58 ± 0.47	89.81 ± 0.86	42.80 ± 0.72	96.93 ± 0.15	0.615 ± 0.023	1.87 ± 0.06
	7.0	41.66 ± 2.04	99.72 ± 0.10	85.07 ± 0.81	97.37 ± 0.12	1.382 ± 0.018	3.05 ± 0.20
	8.0	43.19 ± 1.28	99.85 ± 0.03	87.69 ± 0.62	97.75 ± 0.10	1.483 ± 0.009	3.36 ± 0.05
	9.0	49.80 ± 0.47	99.95 ± 0.02	88.90 ± 0.55	97.92 ± 0.26	1.689 ± 0.017	3.68 ± 0.17
Initial inoculation amount (OD_680_)	0.1	34.94 ± 1.47	98.94 ± 0.06	74.79 ± 1.13	93.73 ± 0.39	1.081 ± 0.025	3.45 ± 0.10
	0.2	44.61 ± 0.74	99.94 ± 0.03	82.38 ± 0.88	94.57 ± 0.35	1.611 ± 0.024	4.16 ± 0.03
	0.4	52.69 ± 0.82	99.99 ± 0.01	89.09 ± 0.37	94.64 ± 0.43	1.699 ± 0.018	5.30 ± 0.10
	0.6	51.25 ± 1.35	99.92 ± 0.05	89.28 ± 0.57	95.30 ± 0.27	1.562 ± 0.011	5.19 ± 0.05

Data are shown as their replicate mean ± SD.

**Figure 5:**
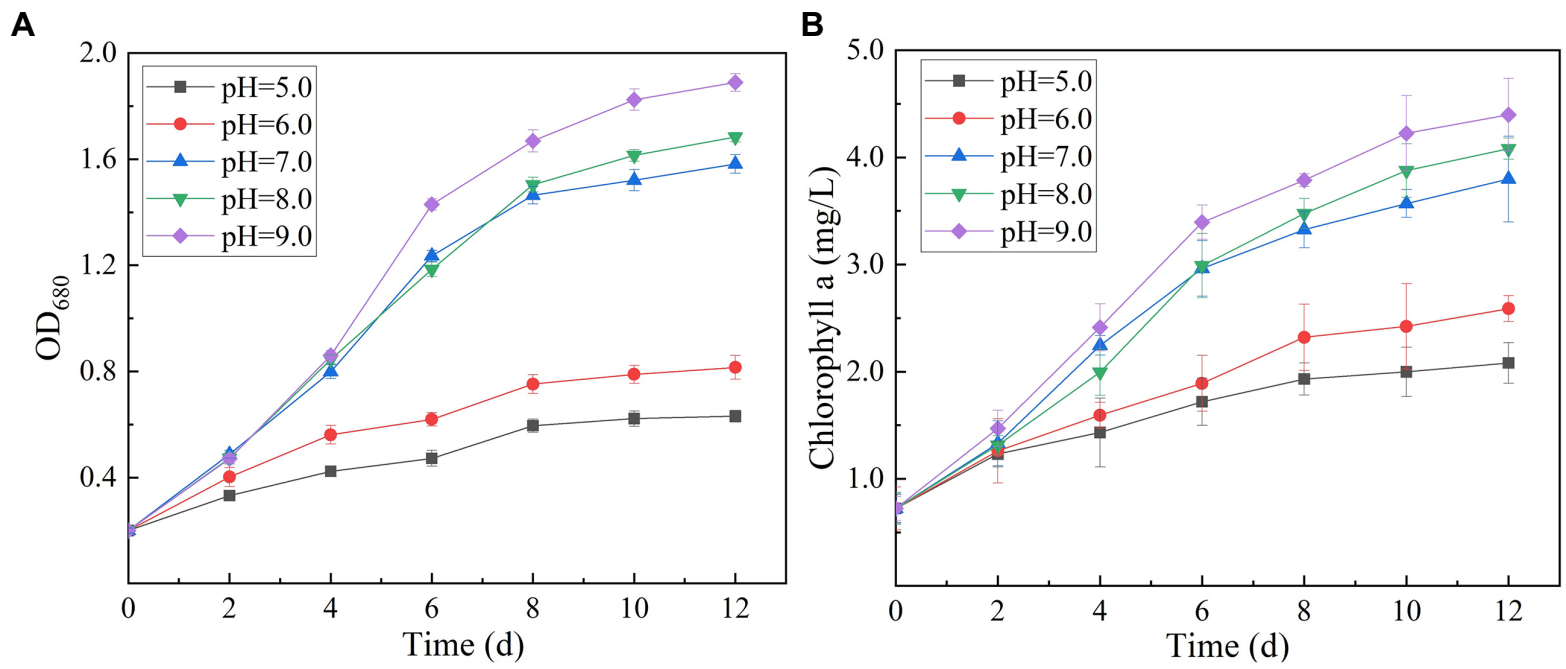
Biomass and chlorophyll a content of algal strain SNN1 at different initial pH (5.0–9.0) of test culture. **(A)** Biomass; **(B)** Chlorophyll a content.

Chlorophyll is a green pigment, which exists in plants, algae, cyanobacteria, and other aerobic photosynthetic organisms. It plays a central role in photosynthesis by absorbing and transmitting light energy (Li and Chen, 2015). Chlorophyll content is an important index reflecting the ability of microalgae to convert inorganic nutrients into organic substances. Variation in culture pH had statistically significant effects on the growth and ultimate biomass accumulation of SNN1 (Figure 5). The results showed that at low culture pH, the growth of SNN1 was inhibited. Among experimental groups with different pH (5.0–9.0), maximum biomass (OD_680_ = 1.889), and Chl a content (4.40 mg/L) was recorded at pH 9.0. With the decrease in culture pH, the biomass accumulation and Chl a concentration decreased sharply, so that at pH 5.0, they decreased by 66.60 and 52.67%, respectively. This reduction in growth can be a result of inhibition of cell division at low pH. Chorvatova et al. (2020) showed that most algae grow and breed in water with high pH value ranging from 7.0 to 9.0, and the optimal pH value is 8.2–8.7. Thus, pH 9.0 is the optimum pH for the growth of SNN1, which strengthens the earlier report that *Chlorella* showed maximum biomass yield under alkaline conditions (pH 9.0–10.0; Khalil et al., 2010). Another study showed that the increase of pH value was helpful to control the pollution of invasive microorganisms (fungi and bacteria; Shahid et al., 2021b). Therefore, the high alkaline environment inhibits the growth of miscellaneous bacteria, which is more conducive to the growth and reproduction of microalgae.

#### 3.3.2. Initial inoculation amount

**Figure 6:**
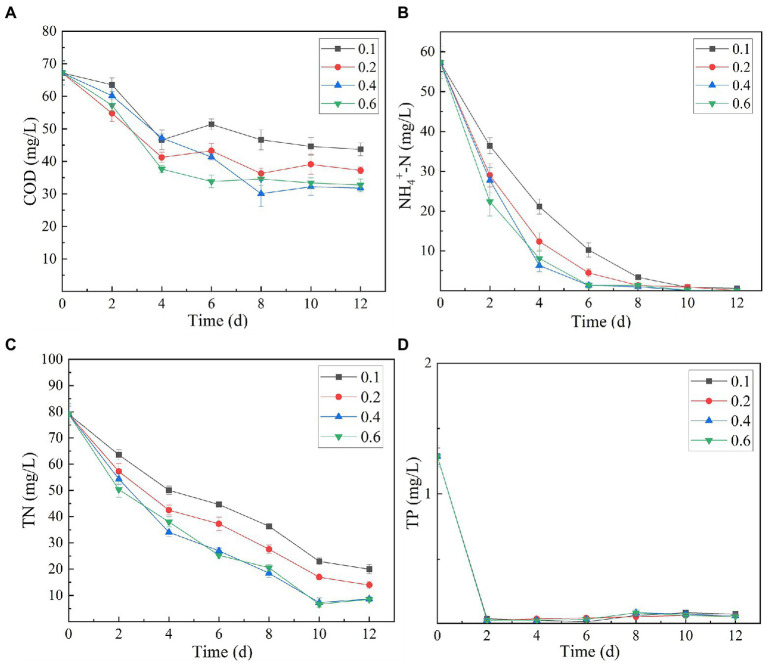
Effect of different initial inoculum quantities (OD_680_) of algal strain SNN1 on its nutrient removal efficiency during 12 days of culture incubation. **(A)** COD; **(B)** NH_4_^+^-N; **(C)** TN; and **(D)** TP.

The analysis of the effect of initial inoculum of OD_680_ in gradient of 0.1, 0.2, 0.4, and 0.6 on treatment of secondary effluent (pH 9.0) showed that the density of initial inoculum had a significant impact on the nutrient removal efficiency of the strains (Figure 6). Under different inoculation concentrations, the NH_4_^+^-N content gradually decreased, and by the end of the experiment (twelfth day), it was totally removed (Figure 6B). TP content was almost completely exhausted on the second day of culture incubation, although its concentration increased slightly at the later stages of the experiment (Figure 6D). This can be explained by the phenomenon that in the later stages of growth, insufficient nutrients cause death of microalgal cells. These dead cells release phosphorus and increase its concentration in the culture. At initial inoculation amount of OD_680_ 0.4 and 0.6, and cultivation for 12 days, SNN1 exhibited the most significant removal effect for COD and TN. The removal rates of COD were 52.69 and 51.25%, and the removal rates of TN were 89.09 and 89.28%, respectively (Figures 6A,C; Table 2). This can be explained with an example of the previous study, which described that at high biomass was beneficial to improve subsequent nutrients and carbon dioxide removal efficiency (Ummalyma et al., 2017). Based on these findings, we suggest that the inoculation amount of 0.4 (OD_680_) is effective for the efficient treatment of secondary effluent by SNN1.

**Figure 7:**
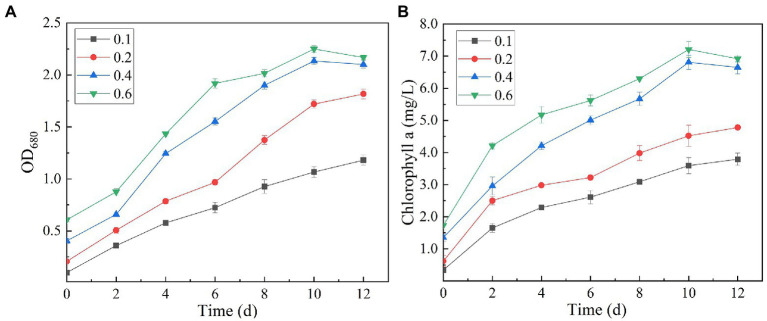
Biomass (OD_680_) and chlorophyll a (OD_665_ and OD_649_) content of algal strain SNN1 under different initial inoculation quantities measured on UV–vis spectrophotometer. **(A)** Biomass; **(B)** Chlorophyll a content.

It can be seen from the figure that the biomass and Chl a content of SNN1 increased with the increase of initial inoculation amount (Figure 7). With the initial inoculation amount of 0.6 (OD_680_), the biomass and Chl a content of SNN1 were 2.168 and 6.92 mg/L, respectively. These findings are supported by previous work, according to which high initial inoculation amounts of microalgae result in higher final biomass of microalgae with a relatively short growth cycle (Mandeno et al., 2005). However, Table 2 shows that biomass and Chl a accumulation of initial OD_680_ of 0.4 were 1.699 and 5.30 mg/L, higher than the 1.562 and 5.19 mg/L with initial OD_680_ of 0.6, respectively. The biomass and Chl a accumulation at initial inoculum of 0.4 (OD_680_) were 8.77 and 2.12% higher than that of inoculum of 0.6 (OD_680_), respectively. This can be explained with an example of the previous study, which described that at higher density of the algal culture, the light transmittance through the culture was reduced due to the mutual shielding of algal cells. This reduction in light transmittance through the culture affected the growth of *Chlorella* in the later stages of the experiment (Wang et al., 2007; Godos et al., 2010). Excessive algal concentration reduces the photosynthetic efficiency of the cells, affecting the growth of microalgae and chlorophyll synthesis (Lau et al., 1995). Moreover, the inoculation amount of 0.4 (OD_680_) can achieve the required biomass faster than the other OD values and reduce the culture cost. Therefore, it is necessary to select an appropriate initial inoculation amount. It is concluded that at the initial inoculation amount of 0.4 (OD_680_), SNN1 effectively removed the nutrients in the secondary effluent and achieved high biomass accumulation.

Whereas, when the initial pH was 9.0 and the initial inoculation amount of algal strain was 0.4 (OD_680_), NH_4_^+^-N, TN and TP in the secondary effluent were 31.79, 0.008, 8.631 and 0.069 mg/L, respectively, after 12 days treatment, the removal rate of NH_4_^+^-N (99.99%) and COD (52.69%) of SNN1 was the highest in the secondary effluent. While the biomass (OD_680_) and Chl a accumulation amounts were also the highest when the initial OD_680_ was 0.4 (Table 2). Therefore, these are the optimum conditions for strain SNN1 to treat secondary effluent efficiently.

### 3.4. Lipid production

**Figure 8:**
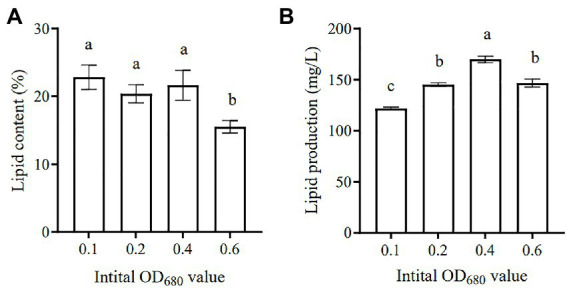
Lipid content **(A)** and Lipid production **(B)** of algal strain SNN1 under different initial inoculation quantities. The values shown were derived from three independent experiments, and error bars represent standard deviations. Data were compared by using one-way ANOVA, with Tukey’s multiple comparison test (*p* < 0.05). The same letter indicates that the difference is not significant and different letters indicate significant differences.

Microalgae are regarded as potential candidates for producing energy (e.g., biodiesel and biogas), as well as coupling with wastewater treatment, which has received much attention in recent years (Unkefer et al., 2017; Shah et al., 2018). Various microalgae species have been identified as having high lipid content, which can be converted into biodiesel, thus providing an alternative source of petroleum-based diesel (Chisti, 2008). Figure 8A lists the summary of lipid content and lipid yield of SNN1 under different initial inoculation amounts used in this study. The lipid content accumulated at the initial inoculation amount of 0.1 was the highest (23.73%). The lipid content decreased with the increase of the initial inoculation amount, as at the initial inoculation amount of OD_680_ 0.6 it reduced to 15.99%.

Studies have shown that the microalgae culture can be divided into two stages: the stage of nutrient sufficiency and the stage of nutrient depletion. In the stage of nutrient sufficiency, the biomass of microalgae increases rapidly, while in the stage of nutrient deficiency, the lipid accumulation increases significantly (Shen et al., 2020). At the later stage of culture, except for the high COD content of each group, NH_4_^+^-N and TP in each experimental group were basically removed. The experimental group with OD_680_ 0.4 and 0.6 exhibited low TN content. But COD and TN in the experimental group with an initial inoculation amount of 0.1 were not completely removed. In contrast, Li et al. (2010) have reported that under nitrogen (2.5 mg/L) or phosphorus (0.1 mg/L) limitation, *Scenedesmus* sp. LX1 could accumulate lipids to as high as 30 and 53% of its algal biomass, respectively. This variation would have been caused by the difference in initial inoculation amount.

In addition, when the initial inoculation amount was 0.4, the lipid production of SNN1 was up to 168.33 mg/L (Figure 8B). This may be due to the high biomass accumulation of strain SNN1, which leads to an increase in lipid production.

## 4. Conclusion

A green microalga with self-flocculating property was successfully separated from the water and mud of Yingxue Lake of Shandong Jianzhu University and identified as *Desmodesmus* sp. SNN1. Compared with other isolated and screened algae strains, SNN1 grows well in the secondary effluent and has higher nutrient removal capacity. It has high potential for wastewater treatment and can be a good option for future research.

Based on the experimental results under different environmental conditions, the optimal culture conditions obtained were as follows: the initial pH of secondary effluent was 9.0, and the initial inoculation amount of algae was OD_680_ 0.4. Under such conditions, it is favored to the production of biomass and chlorophyll a, and the removal of nutrients. The removal effect and biomass were further improved by utilizing these conditions compared with the conditions used for initial screening. The strain has shown promising potential for secondary effluent treatment (removal of 89.09% total nitrogen, 94.64% total phosphorus, 52.69% COD, and 99.99% NH_4_^+^-N) along with a higher and contamination-free growth owing to its alkaliphilic nature (pH 9.0). In addition, SNN1 also possessed a certain lipid production capacity. Under the optimal culture conditions, the lipid content was 20.52%, and the lipid production was 168.33 mg/L. Hence, we can propose that strain SNN1 is a promising microalga, which can be used for treatment of secondary effluent and biodiesel production. This study lays a foundation for the treatment and resource utilization of secondary effluent, and provides pilot scale conditions to be used for large-scale production of microalgae oil.

## Data availability statement

The data presented in the study are deposited in the GenBank repository, accession number OQ198796.

## Author contributions

GC conceived and designed the experiments. PW, YS, YG, RM, WY, ML, XS, and HW performed the experiments. GC and HW contributed reagents, materials, and analysis tools. GC, PW, YS, and RM wrote the paper. All authors contributed to the article and approved the submitted version.

## Funding

This work was financially supported by the National Natural Science Foundation of China (32170396) and the Agricultural Science and Technology Innovation Project of Shandong Academy of Agricultural Sciences (CXGC2022C05 and CXGC2022A22).

## Conflict of interest

The authors declare that the research was conducted in the absence of any commercial or financial relationships that could be construed as a potential conflict of interest.

## Publisher’s note

All claims expressed in this article are solely those of the authors and do not necessarily represent those of their affiliated organizations, or those of the publisher, the editors and the reviewers. Any product that may be evaluated in this article, or claim that may be made by its manufacturer, is not guaranteed or endorsed by the publisher.

## Footnotes

^1^ http://www.ncbi.nlm.nih.gov/blast/

## Supplementary material

Supplementary Figure S1 Phylogenetic trees of algal strains based on 18s rDNA sequences constructed by MEGA 11 software using neighbor-joining method. **(A)** SNN1; **(B)** SNN2; **(C)** SNN4; **(D)** SNS1.
