# Supplementary figures and images for: Advanced treatment of secondary effluent from wastewater treatment plant by a newly isolated microalga Desmodesmus sp. SNN1
Source: Front Microbiol. 2023 Jan 26;14:1111468. doi: 10.3389/fmicb.2023.1111468 (PMC9909749; doi:10.3389/fmicb.2023.1111468)

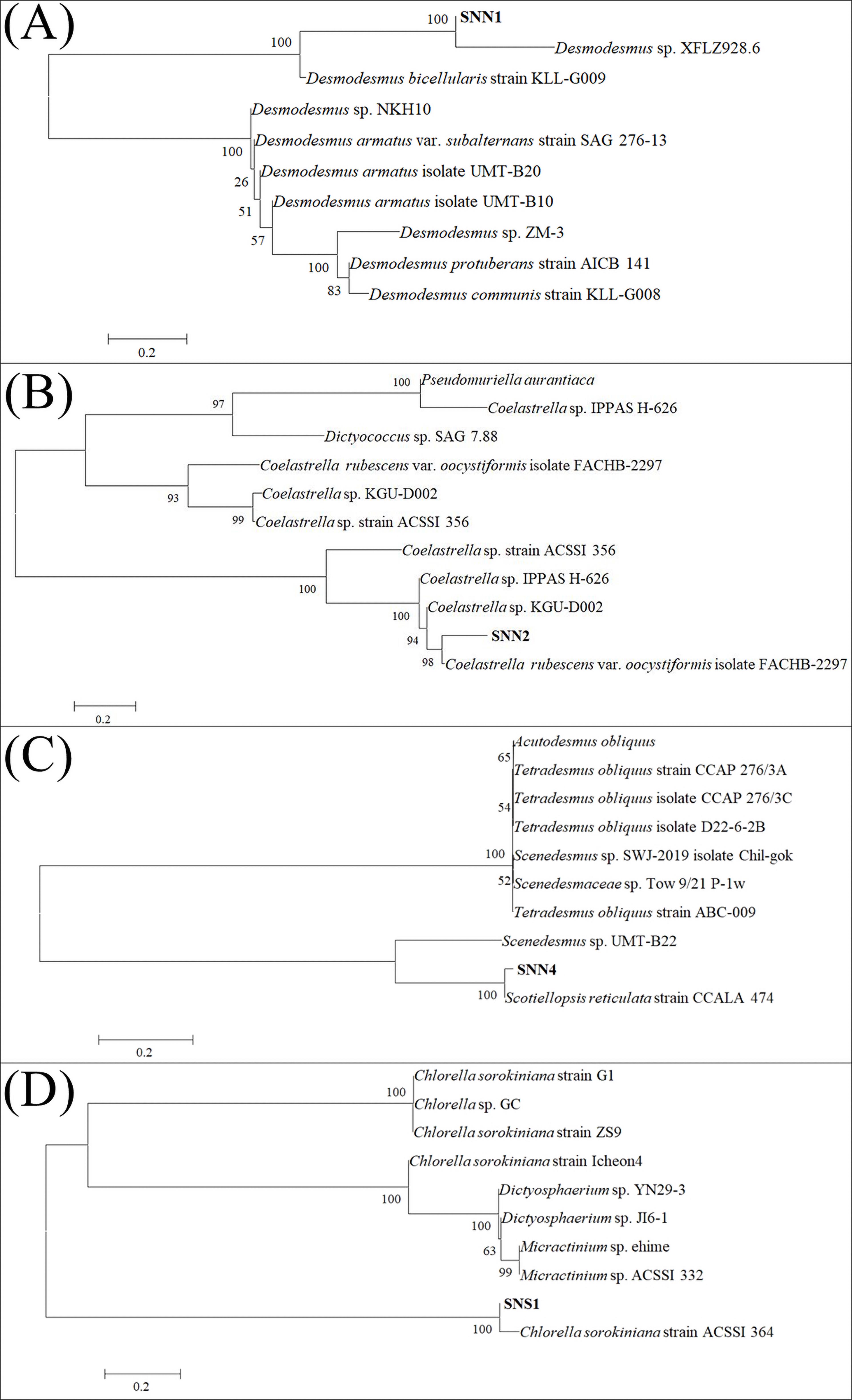

Supplement: Supplementary Figure S1 — Phylogenetic trees of algal strains based on 18s rDNA sequences constructed by MEGA 11 software using neighbor-joining method. (A) SNN1; (B) SNN2; (C) SNN4; (D) SNS1. [file Image_1.jpg]
